# Patient factors associated with SSRI dose for depression treatment in general practice: a primary care cross sectional study

**DOI:** 10.1186/s12875-014-0210-9

**Published:** 2014-12-24

**Authors:** Chris F Johnson, Nadine J Dougall, Brian Williams, Stephen A MacGillivray, Alasdair I Buchanan, Richard D Hassett

**Affiliations:** 1 NHS Greater Glasgow and Clyde, Pharmacy and Prescribing Support Unit, Queens Park House, Langside Road, Glasgow, G42 9TT UK; 2 Nursing Midwifery and Allied Health Professionals Research, University of Stirling, Unit, Unit 13 Scion House, Stirling University Innovation Park, Stirling, FK9 4NF UK; 3 Social Dimensions of Health Institute, University of Dundee, Airlie Place, Dundee, DD1 4HN UK; 4 NHS Greater Glasgow and Clyde, Glasgow City Community Health Partnership, Templeton Business Centre, 62 Templeton Street, Glasgow, G40 1DA UK

**Keywords:** Family practice, Chronic disease, Depression, Antidepressive agents, Benzodiazepines

## Abstract

**Background:**

Antidepressant prescribing continues to rise. Increased long-term prescribing and higher doses are contributing to current growth; however, patient factors associated with the use of higher doses remain unknown. This study’s aim was to investigate patient factors associated with selective serotonin re-uptake inhibitor (SSRI) prescribed daily dose for depression treatment in general practice.

**Methods:**

A stratified sample of low to high prescribing practices were selected. Routine individual patient-level data were extracted one practice at a time: September 2009 to January 2011. Patients included were ≥18 years, and prescribed an SSRI for depression. Logistic regression analysis was undertaken to assess individual predictor variables on SSRI daily dose by standard therapeutic dose versus higher dose, as SSRIs demonstrate flat dose response curves for depression treatment. Predictor variables included: age, gender, deprivation, co-morbidity, smoking status, being prescribed the same SSRI for ≥2 years, and patients’ general practice. For a subgroup of patients a second sub-group analysis included long-term benzodiazepine and/or z-hypnotic (B&Z) as a predictor variable.

**Results:**

Inter-practice SSRI prescribing varied significantly; practice point prevalence ranged from 2.5% (94/3697) to 11.9% (359/3007) of the practice population ≥18 years old; median 7.3% (250/3421) (χ^2^ = 2277.2, df = 10, p < 0.001). Overall point prevalence was 6.3% (3518/52575), with 5.8% (3066/52575) prescribed SSRIs for depression of whom 84.7% (2596/3066) had data for regression analysis. Higher SSRI doses were significantly associated with, in descending order of magnitude, individual practice attended, being prescribed the same SSRI for ≥2 years (Odds Ratio (OR) 1.80, 95% CI 1.49 to 2.17, p < 0.001) and living in a more deprived area (OR 1.55, 95% CI 1.11 to 2.16, p = 0.009). Higher SSRI doses in the B&Z subgroup were significantly associated with individual practice attended, being prescribed a long-term B&Z (OR 2.05 95% CI 1.47 to 2.86, p < 0.001) and being prescribed the same SSRI for ≥2 years (OR 1.94, 95% CI 1.53 to 2.47, p < 0.001).

**Conclusion:**

Higher SSRI doses for depression were associated with practice attended and being prescribed the same antidepressant for ≥2 years. As long-term antidepressant use increases, the use of higher doses may further contribute to prescribing growth.

**Electronic supplementary material:**

The online version of this article (doi:10.1186/s12875-014-0210-9) contains supplementary material, which is available to authorized users.

## Background

Antidepressant prescribing has increased substantially across Europe, USA and Australia over the last 40 years [1]-[5] and has attracted much discussion, debate and concern over the last 40 years [1],[6],[7]. In Scotland it was estimated that 11.3% of the adult population were prescribed antidepressants in 2010/11 [8]. Concerns over the number of people receiving antidepressants and increased prescribing led the Scottish Government in 2007 to set Health improvement, Efficacy, governance, Access to services and Treatment (HEAT) targets to reduce prescribing. These targets were not met due to poor target design and limited knowledge about antidepressant prescribing and use [9].

As elsewhere, the majority of antidepressants are prescribed by general practitioners (GPs) for the treatment of depression [10]-[12] with the rest prescribed for other conditions [13],[14]. Although prescribing continues to increase, there is no clear corresponding increase in depression incidence or prevalence [15],[16]. Increased prescribing has so far been explained at national and local levels by a combination of factors: increased selective serotonin re-uptake inhibitor (SSRI) use [2],[5], increased long-term prescribing [16] and the probable use of higher doses [12],[17]. SSRIs are of particular interest as they account for 53% of all antidepressant prescriptions and 67% of all antidepressant defined daily doses (DDDs) prescribed [8]. DDDs are units of measurement defined by the World Health Organization as ‘the assumed average maintenance dose per day for a drug used for its main indication in adults’. DDDs do not necessarily reflect the recommended or prescribed daily dose but allow a convenient method to compare prescribing volumes between organisations [18].

There are large variations in prescribing between practices which are influenced by practice level characteristics: list size, number of GPs, proportion of female GPs, and population factors including age, gender, deprivation and long-term illness [19]. Depression prevalence is influenced by similar factors with chronic diseases such as diabetes, coronary heart disease, stroke, hypertension, asthma or chronic obstructive pulmonary disease (COPD) increasing individuals’ depression risk [20],[21]. Smoking has also been reported to have a bidirectional relationship with depression and may influence antidepressant response [22]. We were also interested in the influence of benzodiazepines and/or z-hypnotics (B&Zs) due to their negative effects on depression and weak associations with antidepressant prescribing [23]-[25]. The majority of published studies exploring factors relating to prescribing volumes lack patient-level information, such as antidepressant indication and dose [2],[5],[15]-[17],[19].

Although antidepressant doses are one factor which may contribute to current growth and prescribing variations, it remains unclear to what extent individual patient factors influence daily dose. A better understanding of these may enable the development of strategies supporting more appropriate antidepressant prescribing.

Therefore, the aim of this study was to investigate patient-level factors independently associated with SSRI prescribed daily dose for the treatment of depression in general practice.

## Methods

### Setting and design

This cross sectional study is a secondary analysis of routinely available data from a stratified sample of low to high volume antidepressant prescribing general practices in the NHS Greater Glasgow and Clyde (NHSGG&C). NHSGG&C provides healthcare services for a diverse population of approximately 1.2 million people across a varied urban area containing 269 general practices, and in response to the HEAT target committed to further understanding current practice and supporting appropriate antidepressant use [26].

The 269 practices were ranked low to high antidepressant prescribers, by defined daily doses (DDDs)/1000 patients from Prescribing and Information System for Scotland (PRISMS) for year to March 2009. PRISMS is a web-based application providing information for all community dispensed prescriptions, and can be interrogated to provide practice level reports. Ranked practices were then categorised as low (practice 1 to 89: 8,076 to 25,657 DDDs/1000 patients), medium (practice 90 to 179: 25,666 to 34,872 DDDs/1000 patients) and high (practice 180 to 269: 34,886 to 65,409 DDDs/1000 patients) prescribers; practices were recruited from each category with varying characteristics known to influence antidepressant volumes: practice size and deprivation code [19]. Other factors such as patient ethnicity, GP training and country of birth, and practice rurality although known to influence antidepressant volumes [19] were not included due to unreliable data quality and NHSGG&C serving an urban area.

Practices within each prescribing category with a mixture of characteristics were invited to participate in HEAT target service evaluation work through a third party; their local Community Health and Care Partnership (CHCP) prescribing support team. In 2009 NHSGG&C consisted of 10 CHCPs serving populations with varying levels of deprivation. CHCP prescribing support teams serving areas of low to high deprivation were asked to select and approach potential practices for participation in HEAT target service evaluation work. Six CHCPs supported practice recruitment with 12 practices agreeing to participate. Ethical opinion was sought from the West of Scotland Research Ethics Service on the use of anonymised patient-level data for this study; however, the ethics service considered this study to be service evaluation not requiring ethics service approval, although Caldicott Gaurdian approval would be required [27]. Eleven of the 12 practices gave Caldicott Guardian approval to use anonymised patient-level data (Table 1); one medium prescribing practice declined approval to use anonymised data and were excluded. All practices were ‘paper-light’, recording clinical information electronically for >5 years on individual practices’ General Practice Administration System Scotland (GPASS). GPASS was the most widely used general practice system in NHSGG&C at this time.

**Table 1:** **Practice characteristics**

Practice	*ADM volume DDDs/1000 patients (category)	SSRI volume DDDs/1000 patients (%) ^†^	Total practice population ≥ 18 years (female:male)	Number of GPs	^‡^SIMD quintile	Training practice	% patient prescribed an SSRI (number of patients/practice population ≥ 18 years)
1	9576 (L)	6933 (72.4)	3697 (1072:2625)	2	4	No	2.5% (94/3697)
2	18295 (L)	12630 (69.0)	9806 (5327:4479)	5	5	Yes	3.4% (337/9806)
3	20752 (L)	14600 (70.4)	6736 (3601:3135)	6	1	Yes	5.2% (353/6736)
4	28169 (M)	19714 (70.0)	4324 (2262:2062)	5	4	Yes	6.0% (261/4324)
5	29894 (M)	20860 (69.8)	5741 (2964:2777)	4	5	No	8.5% (487/5741)
6	31038 (M)	20967 (67.6)	3421 (1657:1764)	3	4	No	7.3% (250/3421)
7	35490 (H)	25448 (71.7)	3956 (2005:1951)	3	2	No	7.6% (299/3956)
8	41917 (H)	26710 (63.7)	5010 (2493:2517)	6	5	Yes	9.0% (451/5010)
9	44637 (H)	30344 (68.0)	3121 (1653:1468)	3	5	No	8.4% (262/3121)
10	49393 (H)	31885 (64.6)	3756 (1888:1868)	4	4	Yes	9.7% (365/3756)
11	65409 (H)	46309 (70.8)	3007 (1550:1457)	2	5	Yes	11.9% (359/3007)

*From Prescribing Information Systems Scotland (PRISMS) data year to March 2009.

ADM: antidepressant medicines. DDDs: defined daily doses.

Category: Ranked as L – Low, M – Medium and H – High prescribers from PRISMS.

SSRI: selective serotonin re-uptake inhibitors. ^†^% of total antidepressant DDDs/1000 patients.

SIMD: Scottish Index of Multiple Deprivation, ^‡^categorise by practice postcode quintile 1 (least deprived) to 5 (most deprived).

The 11 study practices' patient demographics are similar to 47% (481/1014) of Scottish general practices by urban setting, proportion of patients aged 15 to 74 years old and patient-level SIMD deprivation quintiles. These 481 practices serve 55% (3/5.5 million people) of the Scottish population with 202 of these practices being with in NHSGG&C and serving 1 million people [28].

### Identification of study participants

A single cross sectional data extraction was made for each practice between September 2009 and January 2011. A prescribing support pharmacist used electronic data extraction tools specifically designed and piloted to identify all patients prescribed an SSRI within the previous 3 months, and whether the same patients were prescribed the same SSRI for ≥2 years from individual practices’ GPASS. As current guidelines recommend up to 2 years antidepressant treatment for those at higher risk of relapse [29]-[31], this was considered an appropriate measure of long-term antidepressant use. Patients were included if they were ≥18 years old and prescribed an SSRI to treat depression, including mixed depression anxiety.

The tools simultaneously gathered individuals’ antidepressant prescription information, age, gender, co-morbidities (Read Codes for diabetes, coronary heart disease, stroke, hypertension, asthma or COPD), smoking status and Scottish Index of Multiple Deprivation (SIMD) code derived from each patient’s residential postcode [32]. Co-morbidities and smoking status information was readily available, having been recorded and monitored as part of the general practice General Medical Services contract; Quality Outcomes Framework; details of Read Codes see Additional file 1: Appendix 1.

We were aware of limitations with using depression Read Codes as a marker of antidepressant indication as there is no contractual obligation for GPs to code patients receiving treatment for depression. Read Codes are a standard hierarchical classification system for recording patient medical information in UK primary care [33]. Previous studies highlighted a lack of documented diagnosis [10],[34], and audits in five NHSGG&C practices demonstrated <50% of patients receiving antidepressant treatment for depression were coded for depression. Therefore the primary indication was identified using a combination of electronic GPASS Read Codes and patient encounter information. For a small minority of patients electronic records of antidepressant indications were not available from GPASS therefore individuals’ clinical notes were manually checked for antidepressant indication at the date of initiation by NHS staff before the data set was anonymised. Patients with no clear indication were recorded as indication unknown and excluded.

For a subset of 7 practices benzodiazepine and/or z-hypnotic (B&Z) long-term prescribing data were simultaneously collected, as previous practice level studies indicate an association between antidepressant and B&Z prescriptions [25],[35]. However, data was not collected from 4 practices due to limited staff and time resources. The 7 practices were comparable by urban setting, proportion of patients aged 15 to 74 years old and patient-level SIMD deprivation quintiles to 251 Scottish practices serving 1.5 million people, 124 of these practices being within NHSGG&C. Long-term use was defined as ≥8 weeks continuous use as the majority of B&Zs are licensed for short-term use of 2-4 weeks [13].

### Data operationalisation and statistical analysis

Explanatory variables were included in a statistical model which we hypothised from the literature would influence SSRI prescribed daily dose, and are known to be associated with depression and variations in general practice antidepressant prescribing [19]-[22],[25],[35]. These were individuals’ age, gender, residential SIMD quintile, co-morbidity status, smoking status, being prescribed the same SSRI for ≥2 years, and their general practice. Co-morbidity was categorised into three options, having none, one or ≥2 co-morbidities.

The outcome variable of interest was patients’ SSRI prescribed daily dose, expressed as DDDs, as defined by WHO [18]. For example, a prescribed daily dose of 20 mg or 30 mg citalopram was recorded as 1 DDD or 1.5 DDDs, respectively. The statistical distribution of SSRI DDD data was decidedly ‘non-normal’, and was ‘tooth-like’ with substantial bimodal peaks observed at DDD equivalents of 1.0 and 2.0. As SSRIs demonstrate a flat dose response curve for the treatment of depression with 1 DDD representing a therapeutic dose [18],[29]-[31],[36],[37], the outcome variable of prescribed daily dose was dichotomised as a binary outcome variable of ≤1 or >1 DDD i.e. those with a standard therapeutic dose versus those with a higher dose. Knowing that a DDD equal to 2 was not necessarily twice as effective as a DDD equal to 1, it was decided to adopt a logistic regression model in preference to an ordinal logistic model.

A multi-level model was considered to take account of clustering within practices, however practice level variables were crude and the number of practices were relatively low limiting the meaningful use of the feature of clustering within practices in a statistical model. Very little work has been published to date on the minimum number of clusters required for a multi-level model, however an exploratory analysis done elsewhere suggested there should be at least 10 to 15 clusters [38], therefore with 11 practices the dataset was on the margins of what may be a robust approach. As the practices were not selected at random, and were a stratified sample, fitting practice as random effects variable was ruled out. It was hypothesised that the individual patient-level factors would be more explanatory of the variability in SSRI prescribing than of practice level factors, and that we could retain practice attended as a fixed effect patient-level variable in a pooled practice model, provided the heterogeneity of the coefficients of each explanatory variable was not dramatically different. To test this we ran a logistic regression model for each practice in turn and tabulated variable coefficients with any statistical significance for gender, age, co-morbidities, smoking status, SIMD code derived from patients’ residential postcode and the use of the same SSRI for ≥2 years. We found that practices did not dramatically differ and proceeded to use the statistical model with practice attended as a patient-level fixed effect variable using the pooled patient data from all practices.

Exploratory analysis revealed a curvilinear relationship with age and prescribed daily dose. Different transformations for age were undertaken and although they improved model fit, the model failed to meet statistical assumptions. However by truncating at ≤70 years, these assumptions were met and this upper age limit was retained in the model.

The approach taken was one of a full model fitting all predictor variables we hypothesised from existing evidence which were known to have an effect on antidepressant prescribing. We then used backwards stepwise elimination of variables, in turn, for those which did not achieve a significance level of p = 0.05 to explore what effect was achieved in gaining model parsimony i.e. the best model ‘fit’ with the fewest number of predictor variables. We pre-specified a low significance level of p = 0.05 as a cut-off in eliminating variables in turn as the dataset contained a large number of individuals enabling statistical significance to be more easily achieved. We retained variables greater or equal to p = 0.05 if they improved model fit.

A second logistic regression analysis was also undertaken for the subgroup of patients from 7 practices with data on long-term B&Z prescribing. B&Z data were categorised into a variable with two groups, being prescribed a B&Z long-term or not.

Data were analysed using Stata 11.2.

## Results

Inter-practice SSRI prescribing varied significantly; practice point prevalence ranged from 2.5% (94/3697) to 11.9% (359/3007) of the practice population ≥18 years old; median 7.3% (250/3421) (χ^2^ = 2277.2, df = 10, p < 0.001). The SSRI point prevalence over all 11 practices was 6.3% (3518/52575) of which 67.3% (2369/3518) were female; 5.8% (3066/52575) of the total practice population received an SSRI for treatment of depression (Table 2).

**Table 2:** **Antidepressant indication**

	Number of patients prescribed an SSRI n = 3518 (%)
Depression/Mixed depression anxiety	3066 (87.2)
Anxiety disorder	305 (8.7)
Bipolar	46 (1.3)
Schizoaffective	38 (1.1)
Personality disorder	10 (0.3)
Unknown	18 (0.5)
Other mental health	15 (0.4)
Other general medical	20 (0.6)

Other mental health: insomnia, eating disorders, etc.

Other general medical: neuropathy, menopausal symptoms, irritable bowel syndrome, premature ejaculation, etc.

Significantly higher SSRI doses were prescribed to patients ≤70 years old than those >70 years (mean ± SD), 1.43 ± 0.69 DDDs versus 1.12 ± 0.51 DDDs (Mann-Whitney U test p < 0.001). There was no significant difference in SSRI prescribed daily dose by gender within the age groups.

### Regression analysis

97.5% (2596/2662) of patients ≤70 years had complete data for all predictor variables, and were entered into a logistic regression model (Table 3). We hypothesised an age gender interaction term would be necessary as women live longer than their male counterparts and older age is associated with lower SSRI doses; however, the interaction term was not significant, did not improve model fit, and was left out. All the model assumptions held: there was no evidence of multi-collinearity (no variables were highly correlated >0.8), the link test was correctly specified (hatsq z = 0.90; p = 0.37), and Hosmer and Lemeshow’s goodness of fit test failed to achieve significance (Chi-square (8) = 6.10; p = 0.64). No outliers were excluded for having disproportionate leverage on the model.

**Table 3:** **Patient demographics and independent variables**

	n = 2662	Unadjusted odds ratio (95% CI)	p-value
Mean Age ± SD (range) years	45 ± 13 (18 to 70)	1.00 (0 .99 to 1.01)	0.85
Male (%)	884 (33.2)	1	
Female (%)	1778 (66.8)	1.03 (0.86 to 1.23)	0.734
Deprivation (%)			
SIMD quintile 1(least deprived)	248 (9.3)	1	
SIMD quintile 2	322 (12.1)	1.17 (0.80 to 1.72)	0.41
SIMD quintile 3	167 (6.3)	1.67 (1.08 to 2.58)	0.021
SIMD quintile 4	522 (19.6)	1.38 (0.98 to 1.94)	0.068
SIMD quintile 5 (most deprived)	1364 (51.2)	1.55 (1.11 to 2.16)	0.009
SIMD unknown (not in model)	39 (1.5)		
^†^Co-morbidities (%)			
0	1728 (64.9)	1	
1	665 (25.0)	1.10 (0.90 to 1.33)	0.356
≥2	269 (10.1)	1.18 (0.90 to 1.54)	0.238
Current Smoking Status (%)			
Non-smoker	1581 (59.4)	1	
Smoker	1050 (39.4)	1.13 (0.95 to 1.34)	0.165
Smoking status unknown (not in model)	31 (1.2)		
SSRI use (%)			
ADM for <2 y (%)	1909 (71.7)	1	
Same ADM for ≥2 y	753 (28.3)	1.80 (1.49 to 2.17)	<0.001
Practice (% practice pop.)			
1	82 (2.2)	1	
2	265 (2.7)	1.98 (1.09 to 3.57)	0.024
3	242 (3.6)	1.26 (0.68 to 2.35)	0.461
4	191 (4.4)	3.26 (1.77 to 5.99)	<0.001
5	372 (6.5)	1.50 (0.84 to 2.69)	0.171
6	201 (5.9)	2.69 (1.47 to 4.94)	0.001
7	224 (5.7)	2.20 (1.21 to 4.01)	0.01
8	322 (6.4)	1.81 (1.01 to 3.24)	0.047
9	181 (5.8)	3.80 (2.06 to 7.01)	<0.001
10	302 (8.0)	2.32 (1.29 to 4.18)	0.005
11	280 (9.3)	3.54 (1.96 to 6.38)	<0.001

Odds ratio: unadjusted. CI: 95% confidence interval. SD: standard deviation. SIMD: Scottish Index of Multiple Deprivation. SSRI: selective serotonin re-uptake inhibitor. ADM: antidepressant medicine.

^†^Co-morbidities: Individuals had one or more of the following: asthma, COPD, cardiovascular disease, stroke, diabetes mellitus and/or hypertension.

Higher prescribed daily dose was significantly associated with the following variables in descending order of magnitude by odds ratios: individual practice attended, being prescribed the same SSRI for ≥2 years, and living in a more deprived area (Table 3). There were significant differences between doses for those prescribed SSRIs short-term versus those prescribed the same SSRI for ≥2 years (Table 4), with significant increases observed for all SSRIs except paroxetine and escitalopram.

**Table 4:** **Mean daily doses and differences in short-term and long-term (same SSRI ≥2 years) mean doses**

	ADM <2 years (n = 1909)		ADM ≥2 years (n = 753)		Difference in mean dose (mg) 95% CI	Mann-Whitney U-test ^‡^	All ADMs (n = 2662)	
	Number of ADMs (%) ^†^	Mean dose (SD) mg	Number of ADMs (%) ^†^	Mean dose (SD) mg			Number of ADMs (%)	Mean dose (SD) mg
Citalopram	929 (34.9)	25.8 (12.2)	258 (9.7)	31.2 (14.8)	5.4 (3.6 to 7.2)	<0.001	1187 (44.6)	27.0 (13.0)
Fluoxetine	753 (28.3)	27.2 (12.0)	316 (11.9)	30.6 (14.0)	3.4 (1.6 to 5.2)	<0.001	1069 (40.2)	28.2 (12.7)
Sertraline	147 (5.5)	91.0 (43.7)	76 (2.9)	106.6 (49.2)	15.6 (2.3 to 28.8)	0.019	223 (8.4)	96.3 (46.1)
Paroxetine	35 (1.3)	28.0 (11.8)	67 (2.5)	29.4 (12.7)	1.4 (-3.6 to 6.4)	0.832	102 (3.8)	28.9 (12.3)
Escitalopram	44 (1.7)	15.2 (5.6)	35 (1.3)	15.4 (6.8)	0.2 (-2.6 to 3.0)	0.94	79 (3.0)	15.3 (6.1)
Fluvoxamine	1 (0.0)		1 (0.0)				2 (0.1)	
Total	1909 (71.7)^†^		753 (28.3)^†^				2662 (100%)	

Note: Total mean dose and difference in doses between short-term and long-term use presented as means and SD to aid clarity of actual differences groups.

ADMs: antidepressant medicines. SD: standard deviation.

^†^: Percentage of total antidepressants prescribed to the 2662 patients.

^‡^: Dose distribution for ADM <2 years and ≥2 years compared using Mann-Whitney U-test.

### Long-term B&Z use

Seven practices had data available for a subsequent analysis exploring B&Z association with SSRI prescribed daily dose. 11.8% (190/1610) of the subset were prescribed long-term B&Zs; 1567 (97.3%) had complete data and were included. In this analysis, we found higher prescribed daily dose was significantly associated with the following variables in descending order of magnitude by odds ratios: individual practice attended, being prescribed long-term B&Z and being prescribed the same SSRI for ≥2 years (Table 5). All the model assumptions held: there was no evidence of multi-collinearity (no variables were highly correlated >0.8), the link test was correctly specified (hatsq z = 0.87; p = 0.39) and Hosmer and Lemeshow’s goodness of fit test failed to achieve significance (Chi-square (10) = 3.24; p = 0.92).

**Table 5:** **Patient demographics and independent variables, including benzodiazepines and z-hypnotics**

	n = 1610	Unadjusted odds ratio (95% CI)	p-value
Mean Age ± SD (range) years	46 ± 12 (18 to 70)	1.00 (1.00 to 1.01)	0.432
Male (%)	551 (34.2)	1	
Female (%)	1059 (65.8)	1.17 (0.93 to 1.46)	0.186
Deprivation (%)			
SIMD quintile 1 (least deprived)	93 (5.9)	1	
SIMD quintile 2	153 (9.7)	0.92 (0.50 to 1.70)	0.794
SIMD quintile 3	90 (5.7)	1.75 (0.90 to 3.43)	0.101
SIMD quintile 4	264 (16.7)	1.29 (0.73 to 2.28)	0.373
SIMD quintile 5 (most deprived)	985 (62.1)	1.41 (0.82 to 2.44)	0.213
SIMD unknown (not in mode)	25 (1.6)		
^†^Co-morbidities (%)			
0	1028 (63.9)	1	
1	415 (25.8)	1.20 (0.93 to 1.55)	0.152
≥2	167 (10.4)	1.12 (0.79 to 1.58)	0.533
Current Smoking Status (%)			
Non-smoker	900 (56.6)	1	
Smoker	691 (43.4)	1.16 (0.93 to 1.45)	0.192
Smoking status unknown (not in model)	19 (1.2)		
SSRI use (%)			
ADM for <2 y (%)	1143 (71.0)	1	
Same ADM for ≥2 y	467 (29.0)	1.94 (1.53 to 2.47)	<0.001
Practice (% practice pop.)			
1	82 (5.1)	1	
3	242 (15.0)	1.48 (0.77 to 2.83)	0.241
6	201 (12.5)	2.78 (1.49 to 5.18)	0.001
8	322 (20.0)	1.96 (1.07 to 3.58)	0.029
9	181 (11.2)	4.17 (2.22 to 7.81)	<0.001
10	302 (18.8)	2.60 (1.42 to 4.78)	0.002
11	280 (17.4)	3.62 (1.98 to 6.66)	<0.001
B&Z use (%)			
None	1420 (88.2)	1	
Long-term B&Z for ≥8 weeks	190 (11.8)	2.05 (1.47 to 2.86)	<0.001

CI: 95% confidence interval. SD: standard deviation. SIMD: Scottish Index of Multiple Deprivation. SSRI: selective serotonin re-uptake inhibitor. ADM: antidepressant medicine.

^†^Co-morbidities: Individuals had one or more of the following: asthma, COPD, cardiovascular disease, stroke, diabetes mellitus and/or hypertension.

B&Z: bzodiazepine and/or z –hypnotic.

## Discussion

### Summary of main findings

Higher SSRI doses for depression treatment were statistically significantly associated with the following variables in descending order of magnitude by odds ratios: individual practice attended, being prescribed the same SSRI for ≥2 years and living in a more deprived area. When long-term B&Z prescribing was explored as an explanatory variable in a separate analysis, SSRI doses were found to be statistically significantly associated with: individual practice attended, long-term B&Z prescribing and being prescribed the same SSRI for ≥2 years.

### Strengths and limitations

The use of patient-level data, specifically individuals’ antidepressant dose and indication, enabled this study to overcome limitations of previous studies [2],[15]-[17],[19],[39]. By excluding non-mental health and non-depression SSRI use we identified characteristics associated with individuals receiving higher SSRI doses for the treatment of depression only.

Unlike other antidepressants, SSRIs demonstrate a flat dose response curve for the treatment of depression with higher than standard doses (>1 DDD) being of questionable benefit [29]-[31],[36],[37]. Therefore the use of logistic regression enabled the identification of patient-level variables associated with differences in SSRI standard therapeutic doses versus those on higher doses which are of clinical interest and possibly of more importance in the long-term use of SSRIs.

However this study has some limitations. The cross sectional nature of the study does not permit any analysis of dose progression in time from first starting an antidepressant; dose values captured may be discontinued, reduced or increased soon after data capture. As data capture was staggered from September 2009 to January 2011 the release of updated and new guidance may have influenced prescribing, although the guidance did not advise new changes in antidepressant doses but further highlighted the non-pharmacological management of depression [30],[40],[41], although antidepressant growth has steadily and consistently continued to increase, on average, by 5% per annum since 2004/05 with no clear change in trajectory since the introduction of updated and new guidance [8]. However we cannot rule out that practices at the end of the data collection period may have changed practice in response to the updated and new guidance.

Another possible confounding factor which may have influenced our results were not knowing whether patients took their medicines as prescribed, however only those for whom prescriptions for the SSRIs were issued within the three months prior to data capture were included, and we knew that patients prescribed the same SSRI for ≥2 years were issued with regular SSRI prescriptions. Depression severity and specialist mental health review may also have influenced the use higher SSRI dose, however the majority of patients with depression are diagnosed and treated by their GP without seeing psychiatrists or attending specialist mental health services, and are seen as having milder depressive symptoms [42]. Patient ethnicity is known to be associated with lower practice level antidepressant and B&Z prescribing [43],[44] and inclusion in our analysis would have provided further context to this study; however, patient-level ethnicity data were unreliably and inconsistent which precluded their inclusion in this study.

As this was not a prospective research study, and was completed as part of NHSGG&C service evaluation and ongoing commitment to understanding and evaluating current practice [26], findings may not be generalisable to other areas. However this study’s findings may be of interest to others working in similar urban practices with similar demographics to this population.

### Comparison with existing literature

We found that 6.3% of the adult practice population were prescribed an SSRI; as expected this was lower than previous UK studies which focused exclusively on SSRI and non-SSRI prescribing: 6.9% [10], 8.6% [12] and NHS Scotland’s estimate of 11.3% [8]. The proportion of females and males prescribed SSRIs of 67.3% and 32.7% respectively is consistent with other studies [8],[39], and 87.2% being prescribed an SSRI for depression is slightly higher than 85.4% previously reported in a general practice study including all antidepressants [45]. In comparison 28.3% being prescribed the same antidepressant long-term is lower than previous studies, 47.1% using the same definition of long-term use [12], and 40.6 to 51.4% [16] and 33 to 55% [10] using slightly different definitions. Differences may also reflect GPs’ interest in depression management and optimal care, in managing depression as a long-term condition, and possibly not reducing review frequency as patients continue on longer antidepressant courses [45].

Previous studies indicate that individual patient-level socioeconomic deprivation is significantly associated with early antidepressant discontinuation [39] whereas this study identifies higher deprivation as having a small but significant association with higher prescribed daily doses.

Complex subjective patient and prescriber factors may influence the size of a prescribed daily dose. As patients become ‘experts’ in their condition they become more informed and enabled, making more informed decisions about medicines; possibly voicing their expectations and preferences to use higher antidepressant doses [46],[47]. Although, prescribers may respond to illness severity, and perceived safety and better tolerability of SSRIs [48],[49], as well as ‘pushing the dose’ as GPs have also previously been criticised for prescribing subtherapeutic doses of antidepressants for the treatment of depression [11]. However, an inextricable combination of these known, and other unknown, factors may have contributed to the average SSRI doses for individual drugs in this study being up to 25% higher for <2 years use, and up to 42% higher for those prescribed the same SSRI for ≥2 years, when compared to previous cross sectional studies [11],[50],[51], and although we acknowledge the dose trajectory limitations of this study the routine use of such higher doses will further drive total antidepressant prescription and DDD volumes as SSRI account for the majority of antidepressants prescribed in Scotland [8].

Unexpectedly, in contrast to practice level studies, prescribed daily dose was not associated with co-morbidity [19]. This suggests that practices with a higher proportion of patients with long-term illness treat more patients with antidepressants rather than prescribing higher doses to fewer patients. This study did not find any association between SSRI dose and smoking which is suspected to influence antidepressant response [22]. However, long-term B&Z use was associated with higher SSRI doses which is compatible with previous observations of increased long-term (>4 weeks) B&Z use with SSRIs [35].

### Implications for practice and research

The overarching challenge for current and future practice is continuing support and management for people with common mental health problems, such as depression which is relapsing and remitting, and of a long-term nature. Pragmatically, as long-term prescribing increases [16] and frequency of review decreases with antidepressant duration [45], more consideration should be given to managing depression as a long-term condition. This would enable opportunities to review and optimise care to match individuals’ needs whether that be pharmacological, non-pharmacological, non-medicalised or a combination of these [12],[52].

In line with current guidance [29]-[31],[40] standard medical texts such as the British National Formulary should consider including information highlighting differences in antidepressant dose response effects for the treatment of depression as, unlike tricyclic antidepressants (TCAs), SSRIs have traditionally been prescribed at therapeutic doses [11]. Over the years campaigns [53] and guidelines [29]-[31] have advised prescribers to increase antidepressant doses to achieve better drug response and remission of depressive symptoms. However this advice is appropriate for routine use of TCAs and venlafaxine but not SSRIs, due to their flat dose response curve [29]-[31],[40]; higher doses do not routinely provide better efficacy [29]-[31] but increase the risk of adverse effects such as anxiety and/or insomnia [36],[54]. Such adverse effects may influence combination antidepressant use and/or concomitant B&Z use [35],[55] with regular B&Z use negatively affecting depression and/or anxiety symptoms [23],[24] possibly resulting in SSRI doses being ‘pushed’ further. As 1 in 10 patients in the B&Z subgroup analysis were prescribed long-term B&Zs and an SSRI, and considering B&Zs negative effects, patients prescribed such combinations should be considered a priority for ongoing review and follow up to minimise inappropriate prescribing, and where possible reduce and stop B&Z use.

The difference in short-term and long-term doses raises complex questions such as whether patients are receiving the most effective drug and dose in line with guidance [29],[30]; what is the potential loss of antidepressant efficacy with treatment duration [56],[57] where some patients respond to ‘pushing the dose’ and others dose reduction [56]; whether neuroprogressive changes in depression affect drug response [58], and the challenge of accurately diagnosing depression [34],[59]-[61].

Future research should consider prospective longitudinal studies assessing antidepressant response and outcomes, dose response and changes over treatment lifetime. Qualitative approaches have a role in exploring service user expectations of antidepressant treatment and dose; why GPs ‘push the dose’ and how continuing care is provided. A prospective comprehensive research study, including patient-level and practice-level variables, from a large random sample of general practices is warranted to enable multi-level modeling and further contextualise this study's findings.

## Conclusion

This study has demonstrated that higher SSRI prescribed daily doses for depression are associated with patients’ general practice attended and being prescribed the same antidepressant for ≥2 years. As long-term antidepressant use increases, the use of higher doses may further contribute to prescribing growth. However, the routine use of such higher SSRI doses for depression is not supported by current evidence or guidelines. Therefore, in the short-term, lower prescribing could be achieved via audit and feedback of practice prescribing patterns [62] and GP face-to-face reviews of those already on long-term antidepressants [12] which have both been effective in reducing costs and prescribing volumes.

## Authors’ information

CFJ is a senior prescribing support and antidepressant specialist pharmacist and honorary lecturer in clinical pharmacy.

NJD is a statistician and lecturer in health services research specialising in quantitative studies in mental health.

BW is professor of behavioural and health services research. He is also director of the Scottish Government funded Nursing, Midwifery and Allied Health Professions Research Unit.

SAM is a senior lecturer in evidence synthesis specialising in the assessment of healthcare technologies for depression.

AIB is a clinical governance co-ordinator with a specialist interest in data extraction from clinical systems.

RDH is a senior prescribing & information analyst and pharmacy technician with specialist knowledge and interest in repeat prescribing systems and processes, including general practice computer programs.

## Additional file

## Electronic supplementary material

Additional file 1: **Appendix 1.** Data extraction information and Read Codes [63]. (DOC 74 KB)

## Abbreviations

ADM: antidepressant medicine
B&Zs: bzodiazepines and/or z-hypnotics
CI: 95% confidence interval
COPD: chronic obstructive pulmonary disease
DDD: defined daily dose
GPASS: General Practice Administration System Scotland
OR: odds ratio
NHSGG&C: NHS Greater Glasgow and Clyde
SD: standard deviation
SIMD: Scottish Index of Multiple Deprivation
SSRI: selective serotonin re-uptake inhibitor
PRISMS: Prescribing Information Systems Scotland
