# Supplementary material for: Patient factors associated with SSRI dose for depression treatment in general practice: a primary care cross sectional study
Source: BMC Fam Pract. 2014 Dec 24;15:210. doi: 10.1186/s12875-014-0210-9 (PMC4341873; doi:10.1186/s12875-014-0210-9)
Supplement: Supplementary file 1 — Additional file 1: Appendix 1. Data extraction information and Read Codes [63]. (DOC 74 KB) [file 12875_2014_210_MOESM1_ESM.doc]

Appendix 1 Data extraction information and Read Codes.

| **Condition** | **Code** | **Clinical concept** | **Contract code^†^** |
| --- | --- | --- | --- |
| Depression | E112. | Single major depressive episode |  |
|  | E113. | Recurrent major depressive episode |  |
|  | E118. | Seasonal affective disorder |  |
|  | E11y2 | Atypical depressive disorder |  |
|  | E11z2 | Masked depression |  |
|  | E135. | Agitated depression |  |
|  | E2003 | Anxiety with depression | yes |
|  | E291. | Prolonged depressive reaction |  |
|  | E2B.. | Depressive disorder NEC |  |
|  | E2B1 | Chronic depression |  |
|  | Eu204 | [X]Post-schizophrenic depression |  |
|  | Eu32. | [X]Depressive episode | yes |
|  | Eu33. | [X]Recurrent depressive disorder | yes |
|  | Eu341 | [X]Dysthymia |  |
|  | Eu412 | [X]Mixed anxiety and depressive disorder |  |
|  | Encounters | depression, depressive, depre* |  |
| **Other co-morbidities** | | | |
| Asthma | H33.. | Asthma |  |
|  | H33zz | Asthma NOS | yes |
|  | 21262 | Exlcude if asthma resolved |  |
| COPD | H3… | Chronic obstructive pulmonary disease |  |
|  | H3y.. | Other specified chronic obstructive airways disease |  |
|  | H3z.. | Chronic obstructive airways disease NOS | yes |
|  | H31.. | Chronic bronchitis |  |
|  | H32.. | Emphysema |  |
|  | H36.. | Mild chronic obstructive pulmonary disease |  |
|  | H37.. | Moderate chronic obstructive pulmonary disease |  |
| CVD | G3… | Ischaemic heart disease |  |
|  | G3z.. | Ischaemic heart disease NOS | yes |
|  | G30.. | Acute myocardial infarction |  |
|  | G30z. | Acute myocardial infarction | yes |
| Stroke | G6… | Cerebrovascular disease |  |
|  | G64z. | Cerebral infarction NOS | yes |
| Hypertension | G2… | Hypertensive disease | yes |
|  | G20.. | Essential hypertension |  |
| Diabetes | C10.. | Diabetes mellitus |  |
|  | C10E. | Type 1 diabetes mellitus | yes |
|  | C10F. | Type 2 diabetes mellitus | yes |
| Bipolar illness | E11.. | Manic-depressive psychoses |  |
|  | Eu31. | [X]Bipolar affective disorder | yes |
| Schizophrenia | E10.. | Schizophrenia |  |
|  | E10z. | Schizophrenia NOS | yes |
|  | Encounters | Schizophren* |  |
| OCD | E203. | Obsessive compulsive disorder |  |
|  | Encounters | Compul* |  |
| Anxiety | E200. | Anxiety states |  |
|  | Eu431 | [X]Post - traumatic stress disorder |  |
|  | E29y1 | Post-traumatic stress disorder |  |
|  | E2001 | Panic disorder |  |
|  | E2022 | Panic disorder |  |
|  | E202. | Phobic states |  |
|  | Encounters | phob* |  |
| Eating disorders | E271. | Anorexia nervosa |  |
|  | E2751 | Bulimia nervosa |  |
| Smoking | 137R. | Current smoker | yes |
|  | 137S. | Ex smoker | yes |
|  | 137 L. | Current non-smoker | yes |

^†^Defined by Scottish Clinical Information Management in Practice January 2009 [63]. All practices used NHSGG&C long-term condition templates to consistently record data for contract work.
